# Tunable population dynamics in a synthetic filamentous coculture

**DOI:** 10.1002/mbo3.1324

**Published:** 2022-10-04

**Authors:** Maurice Finger, Ana M. Palacio‐Barrera, Paul Richter, Ivan Schlembach, Jochen Büchs, Miriam A. Rosenbaum

**Affiliations:** ^1^ AVT—Biochemical Engineering RWTH Aachen University Aachen Germany; ^2^ Faculty of Biological Sciences Friedrich‐Schiller‐University Jena Germany; ^3^ Leibniz Institute for Natural Product Research and Infection Biology, Hans‐Knöll‐Institute Jena Germany

**Keywords:** filamentous coculture, fluorescence, online monitoring, oxygen transfer rate, population dynamics

## Abstract

Microbial cocultures are used as a tool to stimulate natural product biosynthesis. However, studies often empirically combine different organisms without a deeper understanding of the population dynamics. As filamentous organisms offer a vast metabolic diversity, we developed a model filamentous coculture of the cellulolytic fungus *Trichoderma reesei* RUT‐C30 and the noncellulolytic bacterium *Streptomyces coelicolor* A3(2). The coculture was set up to use α‐cellulose as a carbon source. This established a dependency of *S. coelicolor* on hydrolysate sugars released by *T. reesei* cellulases. To provide detailed insight into coculture dynamics, we applied high‐throughput online monitoring of the respiration rate and fluorescence of the tagged strains. The respiration rate allowed us to distinguish the conditions of successful cellulase formation. Furthermore, to dissect the individual strain contributions, *T. reesei* and *S. coelicolor* were tagged with mCherry and mNeonGreen (mNG) fluorescence proteins, respectively. When evaluating varying inoculation ratios, it was observed that both partners outcompete the other when given a high inoculation advantage. Nonetheless, adequate proportions for simultaneous growth of both partners, cellulase, and pigment production could be determined. Finally, population dynamics were also tuned by modulating abiotic factors. Increased osmolality provided a growth advantage to *S. coelicolor*. In contrast, an increase in shaking frequency had a negative effect on *S. coelicolor* biomass formation, promoting *T. reesei*. This comprehensive analysis fills important knowledge gaps in the control of complex cocultures and accelerates the setup of other tailor‐made coculture bioprocesses.

## Graphical abstract

Microbial cocultures are used as a tool to stimulate natural product biosynthesis. To provide detailed insight into coculture interactions, we applied high‐throughput online monitoring of the respiration rate and fluorescence of tagged strains of the fungus *Trichoderma reesei* and the filamentous bacterium *Streptomyces coelicolor* on cellulose substrate. This comprehensive analysis allowed us to fill knowledge gaps of complex cocultures, which accelerates the setup of other tailor‐made coculture bioprocesses.

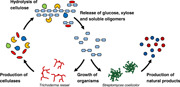

## 1 INTRODUCTION

Cocultivation‐based studies for bioprospecting of natural products and specialized metabolite production are becoming popular in laboratory‐scale fundamental research (Arora et al., 2020; Knowles et al., 2022; Sanitá Lima & Coutinho de Lucas, 2021). Therein, filamentous microorganisms are of great interest, because of their vast biosynthetic potential (Arn et al., 2020; Cibichakravarthy & Jose, 2021; Knowles et al., 2022; Sanitá Lima & Coutinho de Lucas, 2021; Schneider et al., 2018; Zothanpuia et al., 2017). Within these microbes, the genus *Streptomyces* sp. provides most of the antibiotics produced today (Quinn et al., 2020). Although these microorganisms have been broadly investigated, they still offer considerable potential for the discovery of novel biomolecules. This is revealed by the number of silent natural product gene clusters identified in genomic analyses (Belknap et al., 2020; Chung et al., 2021).

The cocultivation approach relies on the paradigm that certain triggers for specialized metabolite production are not likely to be activated under axenic conditions in nutrient‐rich media. Therefore, the synthesis of several biomolecules can only be triggered by signaling compounds or competition with another microbial strain (Boruta et al., 2020; Netzker et al., 2018). This strategy has, for example, successfully been applied in *Streptomyces s*p. for triggering the production of desferrioxamine or the novel antibiotic alchivemycin A (Antoraz et al., 2015; Onaka et al., 2011; Traxler et al., 2013).

Although these biotic induction methods have proven to be fruitful, most studies have been undertaken in a random or empirical manner. Different strains are often simply combined in shake flask cultures, using rich standard cultivation media for *Streptomyces* sp., as reviewed by Boruta (2021), Maglangit et al. (2020), Wakefield et al. (2017), and Yu et al. (2019). However, in the natural environment, nutrients are typically not very abundant and the resulting nutrient availability greatly influences specialized metabolite production (Doull & Vining, 1990; Scherlach et al., 2011). Antibiotic production can often be triggered by the depletion of nutrients to protect the remaining nutrient pool against competing microorganisms (Barka et al., 2016). The natural soil habitat supports this competition, as readily available carbon sources, such as simple sugars, are scarce and most of the available carbon is present in form of recalcitrant lignocellulosic plant material. Therefore, cellulolytic microorganisms play an important role in making this lignocellulosic material available to other microorganisms in the community (Boer et al., 2005). Hence, fermentation of cellulosic substrates by defined cocultures of cellulolytic fungi combined with filamentous soil bacteria represents an interesting strategy to closely mimic the natural situation and induce metabolite and antibiotic production in a controlled and scalable environment.

However, control and scaling of filamentous cocultures using insoluble cellulosic substrates imposes specific analytical challenges for the evaluation of growth and substrate uptake kinetics. As cocultures are highly dynamic and complex, it is difficult to explore their behavior at sufficient temporal resolution using classical offline sampling techniques. The importance of temporal dynamics in cocultures was demonstrated by Bertrand et al. (2014) and Azzollini et al. (2018), who investigated the time‐dependent metabolite profiles of fungal cocultures in miniaturized setups. This showed that the induction patterns of natural products can be very diverse. Many metabolites are enhanced over time until the later stages of the coculture, while others appear only at specific times but are not stable until the end of cultivation. While up‐scaling their cocultures, the authors noticed that the same metabolites could be isolated, but the temporal profile of the induction changed, probably because also the population dynamics changed in the upscaled cultures (Bertrand et al., 2014). Metabolite profiles of cocultures are directly dependent on the underlying population dynamics as demonstrated by Zhang and Zhu et al. (2022), who demonstrated for synthetic cocultures of gut bacteria that the metabolite profiles can even serve as a fingerprint to track the population dynamics of mixed cultures. This highlights the need for noninvasive online monitoring techniques to precisely correlate metabolite profiles with population dynamics. This would further let us establish feedback control strategies to precisely steer population dynamics of mixed cultures and, therefore, allow for rational bioprocess development for cocultures. Recent developments in this field have been extensively reviewed by Schlembach et al. (2021).

As a step toward implementing cellulolytic cocultures as a potential platform for systematic induction of natural products, we highlight in this work how high‐throughput online monitoring allows handling the complexity of cellulolytic filamentous cocultures. Thereby, control strategies for tuning population dynamics were established, to find conditions that favor the formation of specialized metabolites. This is showcased for a model cocultivation system composed of the fungi *Trichoderma reesei* RUT‐C30 and *Streptomyces coelicolor* A3(2), explained in detail in Section 3.1.

## 2 MATERIALS AND METHODS

### 2.1 Microorganisms

Three different organisms were used in this study. *T. reesei* RUT‐C30‐mCherry as well as *S. coelicolor* A3(2) DSMZ 40783 and *Streptomyces coelicolor* A3(2)–mNG. Construction of tagged strains was previously reported (Palacio‐Barrera et al., 2022). Unless stated otherwise, cultivations were inoculated with 10^6^ spores/ml. *S. coelicolor* spores were prepared similarly to Hobbs et al. (1989) and as mentioned in previous works (Finger et al., 2022; Hobbs et al., 1989; Palacio‐Barrera et al., 2022). Briefly, a spore suspension was spread on soy‐flour mannitol agar plates, which consisted of 20 g/L mannitol, 20 g/L soy flour, and 20 g/L agar. The agar plates were incubated for 10 days at 30°C. Thereafter, formed spores were harvested and suspended in deionized water or phosphate‐buffered saline. The spores of *T. reesei* were produced by cultivation in shake flasks with low nitrogen phosphate (LNP) medium (following section) with 30 g/L α‐cellulose. After 7 days, spore formation was indicated by a green coloration. The mycelium of all spore solutions was removed by filtration with a 40 µm cut‐off cell strainer (Corning). Spore concentrations were adjusted in the stock solutions to 10^8^ spores/ml using a particle sizer Coulter counter 4 (Beckman Coulter). *S. coelicolor* spore solutions were stored at 4°C and at −80°C with the addition of glycerol. *T. reesei* spore solutions were stored at −80°C. The stock solutions were vortexed for 30 s before inoculation of the medium.

### 2.2 Media composition

The medium for all cultivations was an LNP minimal medium, which is described also in previous works (Finger et al., 2022; Palacio‐Barrera et al., 2022). For the carbon source, 5 g/L glucose and 30 g/L α‐cellulose were added. The insoluble α‐cellulose (MilliporeSigma former Sigma‐Aldrich) was autoclaved before the liquid medium was added. Medium components were 0.1M 2‐(*N*‐morpholino) ethanesulfonic acid (MES), 3.0 g/L (NH_4_)_2_SO_4_, 0.5 g/L MgSO_4_∙7H_2_O, 0.4 g/L KH_2_PO_4_, 0.3 g/L urea, 0.23 g/L CaCl_2_∙2H_2_O, 0.05 g/L NaCl, 0.25% (v/v) trace element solution, and 0.01% (v/v) Tween‐80. The pH of the medium without trace elements was adjusted to 6.7 with 5 M NaOH. The trace element solution contains 180 g/L citric acid, 16 g/L ZnSO_4_∙7H_2_O, 2.71 g/L CoCl_2_∙6H_2_O, 2.29 g/L Fe_2_(SO_4_)_3_, 2.05 g/L CuSO_4_, 1.60 g/L MnSO_4_∙7H_2_O, and 0.8 g/L H_3_BO_3_. The solutions were sterile‐filtered with a 0.2 µm cut‐off filter (MilliporeSigma).

### 2.3 Cultivations in microtiter plates

Cultivations were performed in 48‐round well plates (Beckman Coulter). For the cultivation conditions, if not stated otherwise, a filling volume of 1 ml, a shaking frequency of 800 rpm, a shaking diameter of 3 mm, and a temperature of 30°C were set. The different factors for control of coculture composition were evaluated in the following manner: For the inoculation ratio, the spore concentrations of the strains were varied, ranging from one to four orders of magnitude below the base spore concentration with 1 = 10^6^ spores/ml. For osmolality experiments, sodium chloride was added to the media with 150, 300, and 600 mM. In this case, the inoculation ratio was set as 1:1. For shaking frequency, parallel cocultures were performed at a shaking frequency of 800 or 1200 rpm. In these last experiments, inoculation ratios were kept within the same order of magnitude.

Two different devices were used for online monitoring: The first device was an in‐house built micro‐respiration activity online monitoring system (µRAMOS) in combination with an in‐house built BioLector system (Flitsch et al., 2016; Ladner et al., 2016). In regular intervals, all wells of the MTP are closed with microfluidic valves for oxygen transfer rate (OTR) determination. The decreasing trajectory of the oxygen partial pressure is measured in the closed well and with a linear approximation, the OTR is calculated. Furthermore, the mCherry signal (excitation: 587; emission; 610 nm) and the green fluorescence signal (excitation: 483; emission: 520 nm) were monitored with a Fluoromax‐4 spectrofluorometer (HORIBA Jobin‐Yvon GmbH). The slit size was 8 nm and the integration time was 900 ms.

The second device was a commercial BioLector I (Beckman Coulter). Filter modules were used for the monitoring of the mCherry signal (excitation: 580; emission: 610nm) and green fluorescence signal (excitation: 480; emission: 520 nm) (Kensy et al., 2009). The fluorescence measurements were performed with a gain of 100.

### 2.4 Offline measurements

Macroscopic pictures of the culture broth were taken with an Epson perfection V700 photo scanner (Epson) or with an EOS 700D photo camera (Canon) after the cultivations were ended. The pH measurements were performed either with a pH‐meter HI 2211 (HANNA Instruments) or MA 235 pH/Ion Analyzer (Mettler Toledo). Extracellular protein quantification was conducted according to the Bradford method, using bovine serum albumin as standard (Bradford, 1976).

## 3 RESULTS AND DISCUSSION

### 3.1 Concept of a cellulolytic coculture for natural product formation

**Figure 1:**
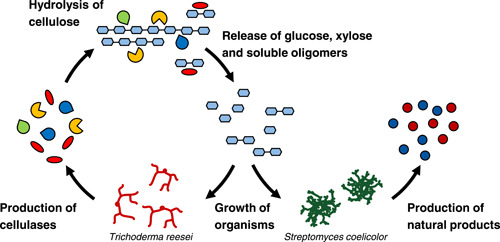
Concept of a bacterial–fungal dependency in a model coculture consisting of the cellulolytic fungus *Trichoderma reesei* and the noncellulolytic bacterium *Streptomyces coelicolor* for the production of natural products from renewable cellulosic material.

A synthetic model coculture system composed of the cellulolytic fungus *T. reesei* RUT‐C30 and the antibiotic‐producing soil bacterium *S. coelicolor* A3(2) (Figure 1) was established in this study. The visible pigments produced by *S. coelicolor* A3(2) serve as easily observable target products, to follow the kinetics of natural compound formation. By growing this coculture on a mineral medium containing cellulose as the main carbon source, a dependency of *S. coelicolor* A3(2) on *T. reesei* RUT‐C30 is imposed, as *S. coelicolor* is unable to consume cellulose. A small amount of glucose is included in the medium to act as a common booster for the initial growth of the two organisms. After an initial nonlimited growth of both partners, ensured by the glucose, further growth on cellulose is only possible with the successful expression of cellulase enzymes by *T. reesei*. It results in a fed‐batch‐like process with carbon‐limited conditions throughout the fermentation, which is known to stimulate antibiotic production (Ates et al., 1997). Similar interactions occur in nature and are reported to lead to natural product formation, for example, antibiotics and pigments, by *S. coelicolor* A3(2) (Aldén et al., 2001; Romero‐Rodríguez et al., 2017).

In this complex system, the initial population dynamics during nonlimited growth on glucose will affect the nutrient partition for cellulase formation and, thus, strongly affect the subsequent population dynamics and natural product formation during growth on cellulose. It is, therefore, of great interest to learn (1) how population dynamics affect natural product formation and (2) how population dynamics can be tuned to maximize natural product formation.

We combine high‐throughput online measurements of OTR and fluorescence to investigate how biotic and abiotic factors influence the population dynamics and production profiles of our model coculture. The OTR holds information about respiration and thereby metabolic activity. It serves as an excellent indicator of substrate consumption dynamics and can be measured noninvasively and online. It has been proven a reliable method for evaluating cellulase formation and cellulose consumption in axenic cultures, as well as defined cocultures (Antonov et al., 2016, 2017; Schlembach et al., 2020). Although the OTR only delivers a sum signal of the coculture respiration activity, the metabolic contribution of each partner can be estimated by comparison to axenic cultures. Further, we have developed a fluorescence‐based procedure for online monitoring of population dynamics in filamentous cocultures, which is applied here (Palacio‐Barrera et al., 2022). Additionally, we previously could show that fluorescence monitoring can help estimate the onset of pigment biosynthesis for *S. coelicolor* (Finger et al., 2022).

Deploying these methods, the influence of inoculation ratio, osmolality, and shaking frequency on the coculture dynamics was studied. First, different inoculation ratios were investigated to understand, which growth kinetics and initial population ratios lead to beneficial interaction between the microorganisms. Different population ratios are also found in natural environments and greatly impact metabolic capabilities (Gao et al., 2021). Then it was analyzed how the population dynamics can be tuned using different osmolalities, and shaking frequencies. Osmolality can alter the growth kinetics and productivity of filamentous organisms (Boruta et al., 2021; Wucherpfennig et al., 2011). Furthermore, shaking frequency and thereby power input is a crucial parameter for filamentous organisms. It can affect the growth rate and morphology of shear‐sensitive filamentous microbes, as well as oxygen availability and physical contact in cocultures. However, to make valid assertions for the observed cocultures, at first the axenic culture behavior had to be characterized by the tested parameters and variables.

### 3.2 Characterization of the axenic culture behavior of the coculture partners

**Figure 2:**
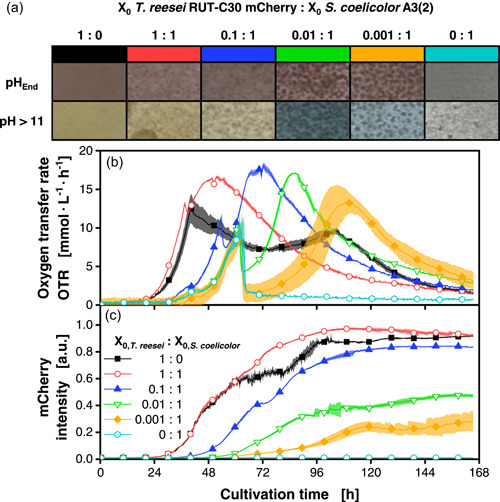
Cocultivations of *Trichoderma reesei* RUT‐C30 mCherry and *Streptomyces coelicolor* A3(2) wild type. Inoculation ratios were varied, as given, relating to the standard inoculation size of *X*_0_ = 10^6^ spores/ml. The spore stock of *S. coelicolor* was prepared with deionized water. (a) Macroscopic pictures of the culture broth after the termination of the cultivation. The pH was changed to a value above 11 to determine the production of the blue pigment actinorhodin (second row in (a)). (b) OTRs. (c) mCherry intensity signals (excitation: 587 nm; emission: 610 nm). For clarity, only a representative amount of data points is indicated by the corresponding symbols in (b) and (c). Culture conditions: 48‐well round well plate, *N* = 3, *V*_L_ = 1000 µl, *n* = 800 rpm, *d*_0_ = 3 mm, *T* = 30°C, LNP medium. LNP, low nitrogen phosphate; OTR, oxygen transfer rate.

As a first step, the growth profiles of the axenic cultures of the partner organisms *T. reesei* RUT C‐30 mCherry and *S. coelicolor* A3(2) wild type were described. The *T. reesei* strain expresses the mCherry fluorescence protein under the control of a synthetic constitutive promoter. This allows precise online monitoring of *T. reesei* biomass in the coculture. The robust correlation of the fluorescence signal‐to‐biomass was demonstrated in our previous study (Palacio‐Barrera et al., 2022). The experiment was undertaken in an in‐house built µRAMOS combined with an in‐house built BioLector system (Figure 2) (Flitsch et al., 2016; Ladner et al., 2016). For a better understanding of interactions in the coculture, especially in regard to cellulose hydrolysis, a chemically defined medium with 5 g/L of glucose and 30 g/L of α‐cellulose as a carbon source was used.

The macroscopic pictures (Figure 2a), taken of the broth at the end of cultivation, indicate a red hue for the axenic *T. reesei* cultivation (Figure 2a, black boxes). The broth of axenic *S. coelicolor* cultivations (Figure 2a, turquoise boxes) presents a white‐to‐grey hue. The coloring of the *S. coelicolor* cultivation can be attributed to unconsumed cellulose. Here, only small pellets of the *Streptomyces* strain can be seen, probably due to the consumption of the initially provided glucose. In contrast, *T. reesei* can produce and secrete cellulases to consume the cellulose in addition to the glucose. Furthermore, *T. reesei* grows in dispersed form and expresses the red‐colored mCherry fluorescence protein.

The online monitoring signals underline these observations. The metabolic activity represented by the OTR (Figure 2b) indicates the growth of the axenic culture of *S. coelicolor* (turquoise line, hexagons) until 62 h. After this time, the signal sharply drops, because of the complete consumption of soluble carbon sources. The actinomycete *S. coelicolor* has at least eight genes encoding cellulases and could theoretically grow on the supplemented cellulose (Bentley et al., 2002). However, Lim et al. (2016) suggest that due to tight expression regulation, no cellulase production and, therefore, no growth can be seen for *S. coelicolor* in a minimal medium with cellulose. Hence, this first OTR peak (turquoise line, hexagons) corresponds solely to the consumption of the initially provided glucose in the medium. In contrast, for *T. reesei* (black line, squares), after the first peak is reached at 40 h, there is no strong drop, but a transition to cellulose consumption. This can be concluded from the OTR integral of approximately 0.92 mol/L, which exceeds the theoretical value achieved by the complete combustion of the initial glucose with 0.166 mol/L. As no carbon source other than glucose and cellulose is present in the medium, the second oxygen consumption peak must be caused by cellulose consumption. *T. reesei* grows faster and has a shorter lag phase with approximately 17 h, compared to *S. coelicolor* with 33 h, in this experiment.

### 3.3 Controlling coculture composition with a variation of the inoculation ratio

With this information, the influence of different inoculation ratios on OTR profiles and *T. reesei* growth was tested. Because the axenic *T. reesei* culture grew faster than the axenic *S. coelicolor* culture, the inoculation density of the faster‐growing fungus was lowered in comparison to *S. coelicolor* to achieve varying coculture compositions. With a highly reduced inoculation ratio for *T. reesei* with 0.01:1 (green line, inverted triangles) and 0.001:1 (yellow line, diamonds), it can be observed that the initial OTR peaks resemble the OTR peak of the axenic *S. coelicolor* cultivation. However, later, there is an additional increase in the OTR in contrast to the axenic culture of *S. coelicolor*. These cultures also exceed the OTR integral of 0.166 mol/L as mentioned above. This implies that *T. reesei* successfully produced cellulases for cellulose hydrolysis and release of additional free sugars in these cocultures. For these conditions, the presence of the pigment actinorhodin was detected, based on a hue change to blue in the final culture broth after the pH is increased to basic values (Figure 2a, green and yellow boxes) (Bystrykh et al., 1996). We suggest that the coculture composition favoring *S. coelicolor* in combination with the slow release of soluble sugars (glucose, cellobiose, and xylose) could trigger pigment biosynthesis, in comparison to the other conditions (Figure 2a) (Kang et al., 1998).

According to the mCherry intensity signal (Figure 2c), *T. reesei* biomass formation took place in all conditions, except for the axenic *S. coelicolor* culture (turquoise line, hexagons). However, due to nutrient competition between *T. reesei* and *S. coelicolor*, different final biomass and, thus, mCherry fluorescence values were reached, depending on the inoculation ratio. The 1:1 and 0.1:1 cocultures reached similar biomass levels of *T. reesei* as the axenic *T. reesei* culture. The slightly lower mCherry signal in the axenic *T. reesei* cultivation (black line, squares) compared to the 1:1 condition (red line, circles) can be explained by enhanced growth on the MTP wall. This also leads to a slight scattering in the mCherry signal after 60 h due to biomass resuspension. In contrast, the 0.01:1 and 0.001:1 cultures show strongly decreased final biomass of *T. reesei*. Therefore, even though the growth of *S. coelicolor* took place in all cocultures as indicated by pellet formation in macroscopic pictures (Figure 2a), more nutrient uptake and biomass formation of *S. coelicolor* was achieved for the 0.01:1 and 0.001:1 conditions. It can be concluded that a strong benefit through inoculation density has to be given to *S. coelicolor*, to successfully compete with the faster‐growing *T. reesei*, and to induce pigment production in these cocultures.

**Figure 3:**
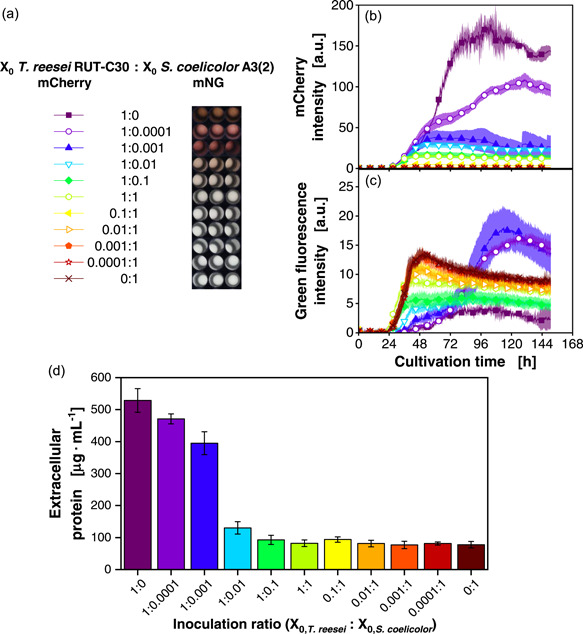
Cocultivations of *Trichoderma reesei* RUT‐C30 mCherry and *Streptomyces coelicolor* A3(2) mNG. Inoculation ratios were varied, as given, relating to the standard inoculation size of *X*_0_ = 10^6^ spores/ml. The spore stock of *S. coelicolor* was prepared with phosphate‐buffered saline. (a) Macroscopic pictures of the culture broth after the termination of the cultivation. (b) mCherry intensity signals (excitation: 580 nm; emission: 610 nm). (c) Green fluorescence intensity signals (excitation: 480 nm; emission: 520 nm). (d) Extracellular protein concentration at the end of cultivation. For clarity, only a representative amount of data points is indicated by the corresponding symbols in (b, c). Culture conditions: 48‐well round well plate, *N* = 3, *V*_L_ = 1000 µl, *n* = 800 rpm, *d*_0_ = 3 mm, *T* = 30°C, LNP medium. LNP, low nitrogen phosphate; mNG, mNeonGreen.

**Figure A1:**
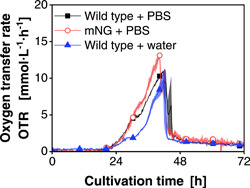
Oxygen transfer rates for axenic cultivations of *Streptomyces coelicolor* A3(2) wild type from Figures 2 and A3 and *Streptomyces coelicolor* A3(2) mNG from Figure 4. Spore stocks were prepared either with phosphate‐buffered saline (PBS) or deionized water. For clarity, only a representative amount of data points is indicated by the corresponding symbols. Culture conditions: 48‐well round well plate, *N* = 3, *X*_0_ = 10^6^ spores/ml, *V*_L_ = 1000 µl, *n* = 800 rpm, *d*_0_ = 3 mm, *T* = 30°C, LNP medium. LNP, low nitrogen phosphate; mNG, mNeonGreen; OTR, oxygen transfer rate.

To validate the conclusions from this experiment, similar cocultures were conducted with an *S. coelicolor* strain tagged with an mNG fluorescence protein as reported by Palacio‐Barrera et al. (2022). This work demonstrates a good correlation of the mNG fluorescence intensity to the dry biomass of *S. coelicolor* and, hence, enables more direct monitoring of individual population dynamics of both organisms. In contrast to the previous coculture experiment, *T. reesei* mCherry grew slower than *S. coelicolor* mNG. This can be recognized by comparison of the respective mCherry and green fluorescence signals (Figure 3b,c) of the axenic cultures. The signals (*T. reesei*—magenta line, squares and *S. coelicolor*—maroon line, cross marks) indicate that the onset of the exponential phase for *S. coelicolor* occurred earlier. The shorter lag phase is also reflected in cocultures ranging from 0.0001:1 to 0.1:1 inoculation ratio (Figure 3b,c, red to yellow lines). The faster growth of *S. coelicolor* appeared to be caused by a change in the spore storage solution from water to phosphate‐buffered saline (PBS). The spore viability in the PBS buffered spore stocks is higher and, therefore, the OTR increases faster. We also confirmed that the tagged *S. coelicolor* strain behaves identically to the *S. coelicolor* wild‐type strain when both are stored in PBS (Figure A1). The faster growth of *S. coelicolor* had a negative influence and led to strong suppression of *T. reesei* growth, due to nutrient competition for initially available glucose. Hence, lower *T. reesei* biomass, as indicated by mCherry signals, and subsequently cellulases were produced in cocultures with inoculation ratios ranging from 0.0001:1 to 1:0.01. This also becomes evident by the comparison of extracellular protein content in cocultures (Figure 3d). However, at inoculation ratios of 1:0.001 and 1:0.0001 (blue and purple bar), similar extracellular protein levels were reached as in axenic *T. reesei* cultures. This indicates efficient cellulase production in these cocultures. In the conditions with 1:0.001 and 1:0.0001, *T. reesei* was able to access the nutrients before *S. coelicolor*, and thereby, it suppressed the initial growth of *S. coelicolor*. The latter becomes evident by the low green fluorescence in the early growth phase of these cultures (Figure 3c, blue line, triangles and purple line, circles). Hence, an interesting switch from competition in the early stage to commensalism in the later stage of the culture can be noticed. Because of the cellulases supplied by *T. reesei*, *S. coelicolor* could access hydrolysate sugars from the cellulose. Thereby, the even higher green fluorescence and, hence, biomass levels than in the axenic cultures of *S. coelicolor* were reached. This was also reflected in the stimulation of pigment production (Figure 3a).

According to the green fluorescence signal (Figure 3c), it can be observed that the *T. reesei* axenic culture displays a low‐intensity green fluorescence signal (magenta line, squares) throughout the cultivation. The recorded green fluorescence is a mixed signal, which in part corresponds to biogenic fluorophores that are found in both partners and secondly, to the mNG green fluorescent protein that is expressed in *S. coelicolor* mNG. Both signals have emissions in the green wavelength region (520 nm). Yet, the mNG labeling strongly enhances the signal and confers a bias towards *S. coelicolor* biomass. Therefore, there is a remarkable difference in the signal from the axenic *T. reesei* cultivation in comparison to the axenic cultivation with *S. coelicolor* mNG (maroon line, cross marks). Thus, despite the effect of autofluorescence, the biomass of the mNG tagged *S. coelicolor* in cocultures can already be evaluated from the uncorrected green fluorescence signal.

**Figure A2:**
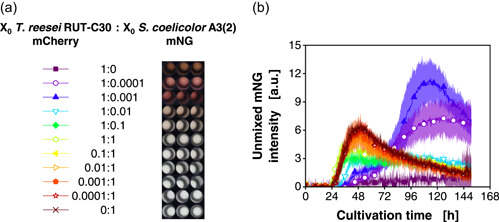
Cocultivations of *Trichoderma reesei* RUT‐C30 mCherry and *Streptomyces coelicolor* A3(2) mNG. Inoculation ratios were varied, as given relating to the standard inoculation size of *X*_0_ = 10^6^ spores/ml. The spore stock of *S. coelicolor* was prepared with phosphate‐buffered saline. (a) Macroscopic pictures of the culture broth after the termination of the cultivation. (b) Unmixed mNG intensity signal from data obtained at channel 1 (excitation: 480; emission: 520 nm) and Channel 2 (excitation: 450; emission: 520 nm). For clarity, only a representative amount of data points is indicated by the corresponding symbols in (a). Culture conditions: 48‐well round well plate, *N* = 3, *V*_L_ = 1000 µl, *n* = 800 rpm, *d*_0_ = 3 mm, *T* = 30°C, LNP medium. LNP, low nitrogen phosphate; mNG, mNeonGreen.

To confirm this interpretation, the autofluorescence bias of the green fluorescence signal was exemplarily separated from the mNG fluorescence through signal unmixing as proposed by Palacio‐Barrera et al. (2022) (Figure A2). Thereby, the green fluorescence that arises from naturally occurring fluorophores was uncoupled from the overall green fluorescence signal. In this manner, a signal exclusively attributed to the mNG fluorescent protein was obtained. Trajectories of the green fluorescence mNG signal (Figure 3c) look very similar to the unmixed signal (Figure A2b) for all cultures with the presence of *S. coelicolor*. In contrast, for axenic *T. reesei* cultivations, the trajectory is flattened when the signal is corrected for autofluorescence (see Supporting Information: Method for additional description).

Overall, successful cellulose‐based co‐cultures are possible, if inoculation ratios are adjusted to allow synchronous growth of the two partners. In contrast, out‐competition is observed when inoculation ratios are mismatched. Hence, the population dynamics highly depend on the timing of the onset of growth, which is not only dependent on inoculum density, but also inoculum viability.

### 3.4 Controlling coculture composition with a variation of the osmolality

Whereas in the first approach the timing of growth onset was varied via inoculation densities and viability of the used spore stock, another approach for steering population dynamics of a coculture could be a direct change of growth rates for each partner. Increased osmolality and water activity are reported to negatively influence the growth rates of fungi such as *T. reesei* (Kredics et al., 2004). However, according to the literature, *Streptomyces* are less affected by increased salt concentrations (Malin & Lapidot, 1996).

**Figure 4:**
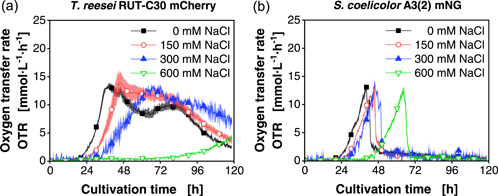
OTRs for axenic cultivations of (a) *Trichoderma reesei* RUT‐C30 mCherry and (b) *Streptomyces coelicolor* A3(2) mNG with varying amounts of added sodium chloride. For clarity, only a representative amount of data points is indicated by the corresponding symbols. Culture conditions: 48‐well round well plate, *N* = 3, *X*_0_ = 10^6^ spores/ml, *V*_L_ = 1000 µl, *n* = 800 rpm, *d*_0_ = 3 mm, *T* = 30°C, LNP medium. LNP, low nitrogen phosphate; mNG, mNeonGreen; OTR, oxygen transfer rate.

For a suitable steering strategy, a parameter, which mainly affects one of the coculture partners, would be highly favorable to reduce the complexity of the system. Therefore, axenic cultivations of *T. reesei* mCherry and *S. coelicolor* mNG were performed by adding increasing amounts of sodium chloride, resulting in a variation of osmolality (Figure 4). Generally, with higher sodium chloride concentrations, the start of the increase in the OTR is prolonged. Especially for *T. reesei* (Figure 4a), the time until the OTR starts increasing for the culture with 600 mM sodium chloride (green line, inverted triangles) is quadrupled, with approximately 72 h compared to 18 h for the control (black line, squares). In contrast, for *S. coelicolor* mNG (Figure 4b) only a delay from 22 to 40 h can be noted.

**Figure A3:**
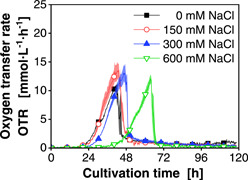
OTRs for axenic cultivations of *Streptomyces coelicolor* A3(2) wild type with varying amounts of added sodium chloride. For clarity, only a representative amount of data points is indicated by the corresponding symbols. Culture conditions: 48‐well round well plate, *N* = 3, *X*_0_ = 10^6^ spores/ml, *V*_L_ = 1000 µl, *n* = 800 rpm, *d*_0_ = 3 mm, *T* = 30°C, LNP medium. LNP, low nitrogen phosphate; OTR, oxygen transfer rate.

Overall, the growth rates of *T. reesei* are more affected in comparison to *S. coelicolor*, as indicated by the substantial drop in OTR increase with higher osmolality. For *S. coelicolor* mNG, the increase remains similar, despite the increase in the sodium chloride concentration. The same effect and OTR trajectory could also be seen for the *S. coelicolor* wild type (Figure A3). Thus, higher osmolality provided a growth advantage to *S. coelicolor* compared to *T. reesei*.

**Figure 5:**
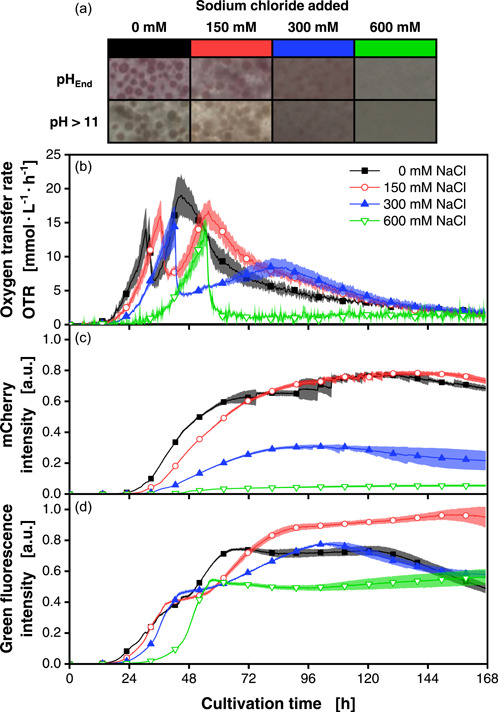
Cocultivations of *Trichoderma reesei* RUT‐C30 mCherry and *Streptomyces coelicolor* A3(2) mNG with varying amounts of added sodium chloride. (a) Macroscopic pictures of the culture broth after the termination of the cultivation. The pH was changed to a value above 11 to determine the production of the blue pigment actinorhodin (second row in (a)). (b) OTRs. (c) mCherry intensity signals (excitation: 587 nm; emission: 610 nm). (d) Green fluorescence intensity signals (excitation: 483 nm; emission: 520 nm). For clarity, only a representative amount of data points is indicated by the corresponding symbols in (b)–(d). Culture conditions: 48‐well round well plate, *N* = 3, *X*_0_ = 10^6^ spores/ml, *V*_L_ = 1000 µl, *n* = 800 rpm, *d*_0_ = 3 mm, *T* = 30°C, LNP medium. LNP, low nitrogen phosphate; mNG, mNeonGreen; OTR, oxygen transfer rate.

The coculture was conducted with a spore inoculation ratio of 1:1 and with the same concentrations of sodium chloride (0–600 mM), as tested before (Figure 5). The macroscopic pictures taken after cultivation (Figure 5a) are similar to the cocultivation pictures in Figure 2a. However, besides the red coloration, which appeared in pH‐untreated samples due to undecylprodigiosin produced by *S. coelicolor* or mCherry of *T. reesei*, no blue pigmentation was observed after a pH increase above 11. A similar effect was reported by Sevcikova and Kormanec (2004), who observed that the production of actinorhodin and undecyprodigiosin was differentially affected at high salt concentrations. Due to unconsumed cellulose, the picture of the condition with 600 mM sodium chloride (Figure 5a, green boxes) remains white‐greyish. For this condition, very low hydrolysis of cellulose and uptake of soluble sugars is indicated by the OTR (Figure 5b). The initial OTR peak (green line, inverted triangles) resembles the axenic OTR peak of *S. coelicolor* in Figure 4b. The slow rise of the OTR afterward is due to the slow growth of *T. reesei* (Figure 5c), along with the production and secretion of cellulases. Overall, a clear and comparable trend to axenic cultures is visible for the change in osmolality. The time until the onset of growth is noticeably prolonged with increasing osmolality (Figure 5b). Additionally, the time between the first and second OTR peak increases, until no second peak is visible anymore for the condition with 600 mM sodium chloride. This can be explained by the coculture composition, similar to that described in Section 3.3. With the increase of added sodium chloride, less *T. reesei* biomass is formed, which is evident in the mCherry intensity data (Figure 5c). The increase in mCherry fluorescence and, thus, biomass formation for *T. reesei* halts after the initial glucose is depleted and rises with the increase in OTR due to cellulose consumption.

**Figure A4:**
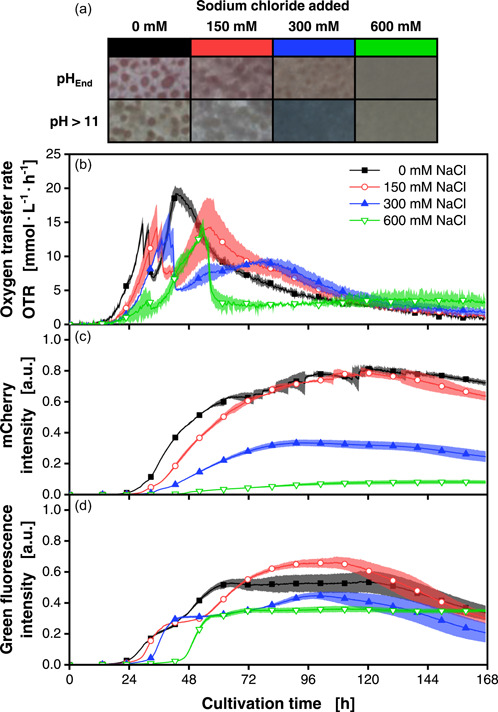
Cocultivations of *Trichoderma reesei* RUT‐C30 mCherry and *Streptomyces coelicolor* A3(2) wild type with varying amounts of added sodium chloride. (a) Macroscopic pictures of the culture broth after the termination of the cultivation. The pH was changed to a value above 11 to determine the production of the blue pigment actinorhodin (second row in (a)). (b) OTRs. (c) mCherry intensity signals (excitation: 587 nm; emission: 610 nm). (d) Green fluorescence intensity signals (excitation: 483 nm; emission: 520 nm). For clarity, only a representative amount of data points is indicated by the corresponding symbols in (b)–(d). Culture conditions: 48‐well round well plate, *N* = 3, *X*_0_ = 10^6^ spores/ml, *V*_L_ = 1000 µl, *n* = 800 rpm, *d*_0_ = 3 mm, *T* = 30°C, LNP medium. LNP, low nitrogen phosphate; OTR, oxygen transfer rate.

The same effect becomes also visible in the green fluorescence signal (Figure 5d), which partly represents the whole coculture growth. However, the mNG labeling confers a bias of this signal for mainly representing the *S. coelicolor* biomass. This was also confirmed when comparing these data with the data of the same experiment conducted with the *S. coelicolor* wild type (Figure A4d). Here, the green fluorescence signals show the same trend but at much lower magnitudes due to the lack of mNG. Overall, these results demonstrate that osmolality is a suitable factor to steer population dynamics by tuning the activity of *T. reesei*.

### 3.5 Controlling coculture composition with a variation of the shaking frequency

**Figure 6:**
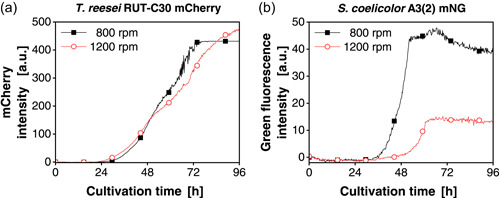
Biomass signal for axenic cultivations of (a) *Trichoderma reesei* RUT‐C30 mCherry and (b) *Streptomyces coelicolor* A3(2) mNG with shaking frequencies of 800 and 1200 rpm. The mCherry intensity signals (excitation: 580 nm; emission: 610 nm) and green fluorescence intensity signals (excitation: 480 nm; emission: 520 nm) were monitored. For clarity, only a representative amount of data points is indicated by the corresponding symbols. Culture conditions: 48‐well round well plate, *X*_0_ = 10^6^ spores/ml, *V*_L_ = 1000 µl, *n* = 800 rpm, *d*_0_ = 3 mm, *T* = 30°C, LNP medium. LNP, low nitrogen phosphate; mNG, mNeonGreen; OTR, oxygen transfer rate.

In the third approach, shaking frequency and, therefore, power input was evaluated as a factor that can influence the population dynamics. The effect of power input on the growth of *S. coelicolor* was previously reported as leading to a change in spore agglomeration (Finger et al., 2022; Tough & Prosser, 1996). Hence, the influence of two different shaking frequencies was tested first on axenic cultivations of *T. reesei* mCherry and *S. coelicolor* mNG (Figure 6).

**Figure A5:**
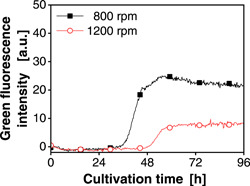
Biomass signal for axenic cultivations of *Streptomyces coelicolor* A3(2) wild type with shaking frequencies of 800 and 1200 rpm. The green fluorescence intensity signals (excitation: 480 nm; emission: 520 nm) were monitored. For clarity, only a representative amount of data points is indicated by the corresponding symbols. Culture conditions: 48‐well round well plate, *X*_0_ = 10^6^ spores/ml, *V*_L_ = 1000 µl, *n* = 800 rpm, *d*_0_ = 3 mm, T = 30°C, LNP medium. LNP, low nitrogen phosphate.

The mCherry intensity signal of both conditions for *T. reesei* (Figure 6a) indicated no effect on the onset of the exponential phase and growth. Therefore, *T. reesei* is mostly unaffected by the tested shaking frequency. In contrast, for *S. coelicolor* mNG (Figure 6b) and *S. coelicolor* wild type (Figure A5) a clear delay in the onset of the exponential phase for cultures shaken at 1200 rpm, compared to 800 rpm, could be noted. Similar to Section 3.4, one coculture partner is mainly affected by the controlling variable. However, here the role is reversed, with *S. coelicolor* growth being more responsive.

**Figure 7:**
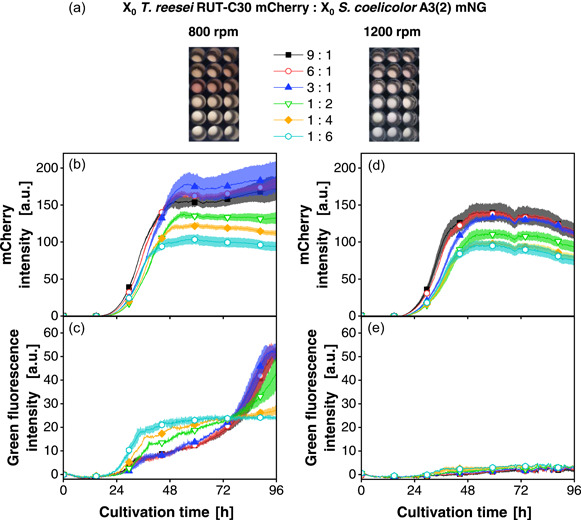
Cocultivations of *Trichoderma reesei* RUT‐C30 mCherry and *Streptomyces coelicolor* A3(2) mNG with shaking frequencies of 800 and 1200 rpm. Inoculation ratios were varied, as given, relating to the standard inoculation size of *X*_0_ = 10^6^ spores/ml. (a) Macroscopic pictures of the culture broth after the termination of the cultivation. (b and d) mCherry intensity signals (excitation: 580 nm; emission: 610 nm) for cultivations at 800 and 1200 rpm, respectively. (c and e) Green fluorescence intensity signals (excitation: 480 nm; emission: 520 nm) for cultivations at 800 and 1200 rpm, respectively. For clarity, only a representative amount of data points is indicated by the corresponding symbols in (b)–(e). Culture conditions: 48‐well round well plate, *N* = 3, *V*_L_ = 1000 µl, *n* = 800 rpm, *d*_0_ = 3 mm, *T* = 30°C, LNP medium. LNP, low nitrogen phosphate; mNG, mNeonGreen; OTR, oxygen transfer rate.

Whether this parameter can be further used to modulate the coculture composition was tested in parallel cocultures at 800 or 1200 rpm. Both partners are expressing fluorescent proteins under the control of a constitutive promoter. A narrow range of inoculation ratios of 9:1, 6:1, 3:1, 1:2, 1:4, and 1:6 within the same order of magnitude from fungi to bacterium were analyzed (Figure 7).

The macroscopic pictures (Figure 7a) indicate coloration, especially for the 800 rpm conditions with an inoculation ratio favoring *T. reesei* (3:1, 6:1, and 9:1). In addition, it can be observed that at 800 rpm (Figure 7c), there is a late increase in the green fluorescence signal for the cocultures with highest *T. reesei* to *S. coelicolor* inoculation ratio. Hence, additional biomass formation of *S. coelicolor* is indicated. For the conditions at 1200 rpm, the picture is different. Despite the gradual increase of green fluorescence (Figure 7e) until 70 h, the total intensity values remained very low. We hypothesize that the higher specific power input under these conditions exerted a growth disadvantage on *S. coelicolor*. Contrasting to the coculture at 800 rpm, where *S. coelicolor* could compete with *T. reesei*, at 1200 rpm the bacteria could not compete for limiting nutrients (soluble carbon source, i.e., glucose or nitrogen).

With the results obtained through OTR and fluorescence online monitoring, we propose that to sustain and control the growth of both partners some prerequisites must be fulfilled: (1) The axenic growth behavior should be well characterized before the cocultivations are conducted. (2) The coculture partner with slower growth should be given an advantage in the coculture, as was presented through inoculation ratio, osmolality, and shaking frequency. The fact that both biotic and abiotic parameters can be used to control the coculture partner activity and that both partners can be independently influenced opens the avenues toward controlled coculture bioprocesses.

## 4 CONCLUSION

In this study, we successfully demonstrated and established a filamentous cocultivation between the cellulolytic fungus *T. reesei* and the model filamentous bacterium *S. coelicolor*. Highly different factors, such as inoculation ratio, osmolality, and shaking frequency, were presented to affect coculture population dynamics. Furthermore, it was shown that after gathering knowledge with high‐throughput online monitoring, the mentioned parameters can be utilized to control the envisioned cocultivation. Monitoring and estimating biomass development with the fluorescence proteins mCherry and mNG were used to effectively determine suitable conditions for the growth of both coculture partners. In addition, the OTR data provided valuable information on metabolic activity, coculture growth, and consumption of cellulose. Slow hydrolysis of cellulose and a coculture advantage for *S. coelicolor* was connected to pigment formation and have to be investigated in further studies. Additionally, the presented approach could be utilized for cocultivation of *T. reesei* with other *Streptomyces* species, using cellulose as the carbon source. These *Streptomyces* strains do not necessarily need to be fluorescently tagged as we could showcase that the OTR in combination with the tagged *T. reesei* strain provides deep insights into the population dynamics. Thereby, the proposed cellulose‐based coculture platform could be explored to systematically unlock the production of novel natural products. Furthermore, analyzing the broth from cultivations with variable population compositions with mass spectrometry could reveal correlations to metabolite profiles and lead to the identification of interesting new natural products, which might require very specific coculture settings to be induced. Such deeper analytical insight would then also lay the foundation for further investigations of the ecological and molecular processes of coculture interaction.

## AUTHOR CONTRIBUTIONS

**Maurice Finger**: Conceptualization (equal); data curation (lead); formal analysis (equal); methodology (equal); visualization (equal); writing–original draft (equal). **Ana M. Palacio‐Barrera**: Conceptualization (equal); data curation (equal); formal analysis (equal); investigation (equal); visualization (equal); writing–original draft (equal). **Paul Richter**: Data curation (equal); formal analysis (supporting); investigation (supporting). **Ivan Schlembach**: Conceptualization (equal); methodology (equal); supervision (equal); writing–review and editing (equal). **Jochen Büchs**: Conceptualization (equal); funding acquisition (equal); resources (equal); supervision (equal); writing–review and editing (equal). **Miriam A. Rosenbaum**: Conceptualization (equal); funding acquisition (equal); resources (equal); supervision (equal); writing–review and editing (equal).

## CONFLICT OF INTEREST

None declared.

## ETHICS STATEMENT

None required.

## Data Availability

Data supporting the conclusions of this study are included within the article and its appendices.

## A.1 | Method—Unmixing of green fluorescence signals

The method reported by Lichten et al. (2014) was used for spectral unmixing of the online fluorescence signal corresponding to the mNeonGreen (mNG) tag. This method was validated and adapted for cocultures by Palacio‐Barrera et al. (2022). Online green fluorescence signals of the mNG tag were unmixed using data of two channels: Data of Channel 1, corresponds to a green fluorescent protein (or GFP‐like) dominated signal (excitation: 480; emission: 520 nm). Data of Channel 2, corresponds to an autofluorescence‐dominated signal (excitation: 450; emission: 520 nm), which is mainly attributed to the biogenic fluorophore riboflavin. Measurements were performed with a gain of 100. A detailed description of method adaption can be found in the work of Palacio‐Barrera et al. (2022).

## A.2 | Discussion—Unmixing of green fluorescence signals

It can be observed that the trends for the unmixed signal (Figure A2) of all traces are very similar when compared to the green fluorescence signal obtained from Channel 1 and portrayed in Figure 3b,c. The fluorescence values are lower since here only the signal that comes from the mNG tag is considered. Herewith, there is a drop of the unmixed fluorescence after 52 h in the axenic culture of *S. coelicolor* mNG as well as in the cocultures with a low *T. reesei* to *S. coelicolor* ratio (brown to green lines). As the mentioned cocultures were unpigmented, we hypothesize that after 52 h there was either biomass death or proteolytic degradation. Likewise, we suggest that the coculture production of fluorescence proteins was discontinued very early, and photobleaching probably occurred. The mentioned drop in unmixed fluorescence is not pH related. The pKa of mNG is 5.7 and, in these cases, the final pH of the broth was above 6.7 (Table A1). In contrast, for the cocultures with the two highest *T. reesei* to *S. coelicolor* ratios, which are highly pigmented, a drop in the unmixed signal is visible at the end of the culture. We suggest that the onset of pigment formation is the main cause of this drop, as already reported by Finger et al. (2022) and Palacio‐Barrera et al. (2022). This exemplary process of unmixing the mNG fluorescence signal as a specific biomass measure for *S. coelicolor* from the green autofluorescence signal shows for this coculture that the overall green autofluorescence signal also gives a good relative account for the biomass of *S. coelicolor* (compare Figure A2b with Figure 3c). Since it was not possible in all used Biolector devices to measure two independent green fluorescence channels simultaneously as is required for applying the unmixing process, we only present the total green fluorescence as a relative signal for *S. coelicolor* biomass for all other experiments of this article.

**Table A1:** Final pH of broth for *T. reesei* mCherry: *S. coelicolor* mNG cocultures and axenic controls

*T. reesei* mCherry: *S. coelicolor* mNG	Final pH
1:0	5.81 ± 0.01
1:0.0001	5.78 ± 0.02
1:0.001	5.90 ± 0.01
1:0.01	6.74 ± 0.02
1:0.1	6.75 ± 0.01
1:1	6.73 ± 0.01
0.1:1	6.75 ± 0.00
0.01:1	6.74 ± 0.00
0.001:1	6.74 ± 0.01
0.0001:1	6.72 ± 0.00
0:1	6.74 ± 0.01

Abbreviation: mNG, mNeonGreen.
